# A Video-Observed Treatment Strategy to Improve Adherence to Treatment Among Persons Who Inject Drugs Infected With Hepatitis C Virus: Qualitative Study of Stakeholder Perceptions and Experiences

**DOI:** 10.2196/38176

**Published:** 2023-06-02

**Authors:** Alison Karasz, Krupa Merchant, Julia Arnsten, Judith Feinberg, Arthur Y Kim, Paula J Lum, Melissa Diane McKee, Shruti H Mehta, Paul Meissner, Brianna L Norton, Kimberly Page, Irene Pericot-Valverde, Reena Singh, Ellen Stein, Lynn E Taylor, Judith I Tsui, Katherine Wagner, Alain Litwin

**Affiliations:** 1 University of Massachusetts Chan Medical School Worcester, MA United States; 2 Northwestern Memorial Hospital Chicago, IL United States; 3 Montefiore Medical Center Bronx, NY United States; 4 West Virginia University School of Medicine Morgantown, WV United States; 5 Harvard Medical School Boston, MA United States; 6 University of California, San Francisco San Francisco, CA United States; 7 Johns Hopkins University Baltimore, MD United States; 8 University of New Mexico Albuquerque, NM United States; 9 Clemson University Greenville, SC United States; 10 University of Rhode Island Providence, RI United States; 11 University of Washington School of Medicine Seattle, WA United States; 12 Prisma Health/Clemson University Greenville, SC United States

**Keywords:** mHealth, video-observed therapy, directly observed treatment, hepatitis C virus, persons who inject drugs, antiviral, adherence, HCV, opioid treatment, mobile health, adherence behavior, behavior intervention, telemedicine, digital health, perception, therapy, treatment

## Abstract

**Background:**

Direct-acting antiviral medications have the potential to eliminate the hepatitis C virus (HCV) epidemic among people who inject drugs; yet, suboptimal adherence remains a barrier. Directly observed treatment (DOT), an effective strategy for optimizing adherence, has been frequently implemented in opioid treatment programs but less commonly in community health settings due to the heavy burden of daily visits. An alternative is video-observed therapy (VOT), which uses mobile health technology to monitor adherence. VOT has not been widely studied among people who inject drugs with HCV.

**Objective:**

This qualitative study, part of a larger implementation evaluation, investigates stakeholder perceptions and experiences with VOT in Project HERO (Hepatitis C Real Outcomes), a multisite pragmatic trial testing treatment delivery models for people who inject drugs with HCV. Our goal was to understand the potential barriers and facilitators to the implementation of the VOT technology.

**Methods:**

Qualitative interviews were conducted with 27 Project HERO study staff and 7 patients. Interviews focused on perceptions and experiences with the VOT app and barriers and facilitators to implementation. Team meeting minutes over the first 2 years of the project were transcribed. A coding system was developed and applied to the data. We summarized thematic data and compared participant perceptions to generate a close understanding of the data.

**Results:**

Frequent barriers to VOT included mechanical failure, stolen or lost phones, and a steep learning curve for participants and study staff. In sites with older and less technically skilled participants, staff found it difficult to implement the VOT app. Research staff found that the routine monitoring of app use led to closer engagement with participants. This was both a benefit and a potential threat to the validity of this pragmatic trial. Patient participants reported mixed experiences.

**Conclusions:**

VOT may be a useful alternative to DOT for some patients, but it may not be feasible for all. Significant staff involvement may be required.

## Introduction

Hepatitis C virus (HCV) is highly prevalent among people who inject drugs [1-5]. The advent of direct-acting antivirals has the potential to halt the HCV epidemic and dramatically reduce the public health burden of the disease. Yet, despite the availability of these new highly efficacious regimens, HCV treatment uptake in this group has been limited by barriers at the individual, health system, social, and economic levels [1,6-8]. Recent data suggest that persons who inject drugs can be successfully treated, with many studies showing high rates of sustained virologic response. Yet, suboptimal adherence remains a barrier for some patients—especially those experiencing social and economic barriers [9-13].

The HERO (Hepatitis C Real Outcomes) study was designed to evaluate HCV treatment delivery models to improve adherence and treatment engagement to provide information on treatment outcomes and reinfection rates among persons who inject drugs who are treated with new generation direct-acting antivirals [14]. Funded by the Patient-Centered Outcomes Research Institute, HERO was a pragmatic, multisite randomized trial implemented in 8 US cities. HCV-infected individuals who reported injecting within the previous 90 days were recruited and randomly assigned to 1 of 2 delivery models: patient navigation or directly observed therapy (DOT). Treatment was provided at 2 types of sites: opioid treatment programs (OTPs) and non-OTP sites—a broad category that included community primary clinics and specialty clinics (infectious disease, HIV).

DOT has been the public health standard of care for tuberculosis treatment since the early 1990s and is a standard practice for people receiving methadone in OTP settings [15]. DOT for HCV treatment has been studied in OTP settings [16-18]. However, DOT is not common in community health settings, due to the heavy burden of daily visits on patients and staff. An alternative to traditional DOT is video-observed therapy (VOT) using mobile health (mHealth) technology.

VOT enables patients to use a smartphone or other video or computer equipment to record and upload videos of medication ingestion. Synchronous versions (in which medication ingestion is viewed in real time) and asynchronous versions (which allow for later review) are available. Although VOT holds promise for the treatment of various disease conditions, it has been used, to date, mainly in tuberculosis treatment [19-22]. A meta-analysis of studies comparing standard DOT with VOT for patients with tuberculosis found that the 2 approaches achieved comparable adherence [23]. A recent synthesis of the literature through 2018 comparing DOT and VOT found high rates of patient adherence and acceptability with VOT, along with lower costs [24]. A trial in the United Kingdom comparing VOT to DOT found that patients assigned to the VOT arm achieved a higher rate of successful observations [19], as did a similar trial in Moldavia [25].

Despite these promising results, however, many studies describe important barriers to implementation. VOT is less resource intensive than DOT but still requires intensive staff engagement [26]. At the patient level, social and economic barriers may prevent uptake. The most common of these is low phone ownership [27,28], lack of technical skills, and a lack of internet access [29,30].

This study is the first to examine patient and staff experiences of VOT among active substance users infected with HCV. In the HERO study, OTP participants randomized to the DOT arm received standard DOT. Those randomized to DOT in non-OTP settings were given a choice of DOT or modified DOT via smartphones (VOT). Each VOT participant was provided with a smartphone, preloaded with the EMOCHA VOT app and issued HCV medication in blister packs weekly for 12 weeks. Participants who wished could use their own smartphones, loaded with the app. The participants received brief training on how to use the app, with follow-up training and support provided on an as-needed basis. Participants recorded themselves taking their daily medication and submitted the video via the app. Research staff documented adherence—defined as the ingestion of medication—by reviewing videos through a web-based platform. The videos were required to show the ingestion of the pill by the participant. If a participant did not submit a video by the required time, they were contacted by research staff within 72 hours.

This study evaluated the implementation of the VOT intervention from the perspective of research staff and patients, drawing on data from a larger, ongoing implementation evaluation of the HERO study. The goal of this study was to understand acceptability among stakeholders of the VOT intervention, strategies for implementation, as well as barriers and facilitators to implementation.

## Methods

### Sample

The study was conducted during 2017-2018. To gauge a broad diversity of views, the sampling plan included stakeholders in key project roles. Accordingly, 27 study staff were included: 9 site principal investigators, 11 project directors, and 7 research assistants. Study staff recruited 7 patient participants who had participated in the HERO VOT intervention. Recruitment of participants for interviews was challenging, due to the difficulty of following up with participants after the end of the active treatment phase. Although the goal was to recruit at least 1 VOT participant at each of the 8 HERO sites, 1 site was unable to do so. An early review of the interview transcripts generated from patient interviews suggested an acceptable level of “data saturation”—the point at which, in the opinion of the analysis team, new data is not providing new analytic insights. Consequently, further interviews were deemed unnecessary.

### Analysis

The implementation evaluation team (AK, RS, KM, and MDM) developed a qualitative interview guide for study staff that focused on implementation experiences and perceptions (Multimedia Appendices 1 and 2). Study staff and participants completed oral informed consent procedures. Interviews were conducted by telephone, taped, and transcribed. Patient participants received a US $15 honorarium for their participation. To further capture ongoing perceptions of the intervention among project staff, we also assembled transcripts of discussions of VOT from the “minutes” taken from research staff meetings between 2016 and 2018. These transcripts were included in the qualitative data set.

Interview and meeting minute transcriptions were uploaded into NVivo (QSR International), a qualitative data analysis program that facilitates the rapid organization and retrieval of thematic data. We developed a preliminary coding scheme for this analysis. Preliminary codes included implementation processes and strategies, barriers and facilitators to implementation, and perceptions or experiences of the VOT intervention. The initial coding scheme was applied to a subset of data by the first and third authors. The coding scheme was subsequently revised and reapplied to the data in an iterative fashion until it was deemed sufficiently parsimonious and comprehensive (Multimedia Appendix 3). At this point, the entire data set was coded. We conducted a descriptive analysis, summarizing and describing key themes in the data.

### Ethics Approval

This study was approved by the Committee on Clinical Investigations at the Albert Einstein College of Medicine (approval number: 2015-5723).

## Results

### The Decision to Implement VOT

Analysis of the transcripts of project meetings helped to clarify the decision-making process regarding the transition to VOT, which had not been part of the original protocol. The original HERO protocol specified in-person DOT at both OTP and community health center sites where HERO patients received their treatment. Initial conversations, before the beginning of the study, had suggested that this might be feasible at some primary care sites. Yet, as the study began, it became clear that staff at the busy health care centers, who received no compensation for providing additional services, lacked the resources to conduct DOT for HERO participants.

Following conversations among study staff, a decision was consequently made to offer VOT as a more feasible option to DOT arm patients receiving treatment in CHCs. These participants were given a choice: VOT via smartphone with daily videos or conventional DOT 3 times per week, with the remaining doses consumed at home. The vast majority of participants chose VOT.

In other studies, reported in the literature, VOT has been implemented with patients owning their own phones. As described above, this requirement poses a serious challenge in settings and populations in which phone ownership and resources to pay for “minutes” is rare. In the HERO study, the company providing the VOT app also provided phones for patient use. Participants with smartphones were given the choice of using their own phones or a company phone. To prevent the expense entailed by the personal use of company phones, the company restricted usage to the intervention only, creating a lock on the phones in an attempt to prevent participants from making personal calls or using data. In practice, however, some participants were able to circumvent the locks and use the phones to make personal calls.

The next challenge was to consider who would provide VOT video adherence monitoring. VOT adherence monitoring includes reviewing videos within a 72-hour period and contacting participants when videos were missed to provide adherence support. The time commitment for clinical staff is lower than that required for DOT but may be prohibitive in many settings. As the HERO project entered its initial recruitment phase, recruitment sites were expanded to include several types of non-OTP sites, including infectious disease and HIV clinics. It became clear quite quickly that clinicians at non-OTP sites were unable or unwilling to provide VOT. Consequently, HERO research staff took on the tasks of video monitoring and follow-up.

As a pragmatic trial, HERO was designed to deliver its interventions under conditions closely approximating those of the real world [14,31], with the goal of maximizing external/ecological validity and the generalizability of results. Among HERO leadership, the question arose of how much support HERO staff should provide. If staff did not monitor participants carefully, this could threaten the fidelity to DOT. Early on in the process, 1 research team member asked:

Patients need to be contacted if videos are not submitted, whether it be on a weekly or biweekly basis, to ensure proper adherence. How can we call it DOT if we do not adhere to this?

Minutes, 2016

The manual of procedures, as ultimately developed, included detailed guidance to research team members on the schedule for participant contacts. When a dose was missed, ideally, research staff were to contact participants on the same day. If that was not possible, they were to reach out to patients at least weekly in the event of missed doses. As is described below, the “missed video” call often evolved into an occasion for research staff to offer various kinds of support and services. Arguably, such close monitoring by research staff posed a threat to the “real world” validity of the study.

### Implementing VOT

Feedback regarding the start-up phase for VOT was mixed. Lost, stolen, and broken phones were a major issue. Data provided by EMOCHA indicates that, of 135 telephone numbers provided to the 8 HERO sites, approximately half of the phones were lost or stolen, including 16 broken and 49 lost or stolen phones. At 1 early meeting, a frustrated project director reported that, of 12 phones issued to the site initially, 3 were broken and 6 were lost or stolen. Participants reporting lost or stolen phones received replacements. Among patient participants in this study, several reported losing their phones during periods of heavy drug use. One participant dropped out of treatment because he was ashamed to acknowledge to the project staff that he had lost his phone.

I went on a binge, and I just lost track of it and lost the phone that I had to record myself with, and I was embarrassed about it and didn’t call [the project director]. I should have.

Participant 2

Another frequently reported problem was the misuse of phones.

The issue that we’ve been having... is we just got an e-mail from the manufacturer of the phone app that one particular patient has been utilizing a lot of data on the phone, which is one indication that he’s using the phone for other purposes.

Project director

In addition to broken phones, technical malfunctions were a common problem. On several occasions, patients could not use their phones when outside a Wi-Fi setting, presumably because the data function was not working. When patients used their own phones, the app tended to freeze their phones. To address these issues, EMOCHA provided considerable technical support, including sending a representative to the site that was having the most trouble.

Once technical glitches were worked out, concerns shifted to the difficulties caused by the lack of technical savvy among some participants.

Some of our participants that we had are not fluent in technology. Never owned a smartphone before. We’re talking about guys who have never owned a cell phone before, don’t know how to work a computer. Asking them to remember a password, a username, to log into this phone, to open up a certain app, to type in their user word, to type in their password that shows up only in asterisks so they can’t even tell if they’ve made a typo, and then going through and recording themselves taking a video every day, it was just awful. I’d spend forever trying to teach . . . people how to use the phone and we’d never ever get a video. . .

Project director

Study staff noted that the app worked better for patients who had their own phones—not so much because of technical problems associated with the app but because participants who owned phones differed from those who did not. Many staff reported that participants with smartphones were younger and more tech savvy. Even more importantly, phone owners tended to experience fewer social and structural barriers to participation, such as deep poverty and homelessness. Some participants seemed pleasantly surprised at how easy it was to use VOT:

They explained it pretty well. . . like for using the video to—using my phone take a video to send in every day was a little cumbersome, but I did that no problem. And they were really good about monitoring and, you know, if I didn’t send in a video on time, they would remind me. It was a breeze.

Participant 1

At some sites, most patients were able to figure it out with the help of coaching from the research staff:

I would say once they got a grasp of it, they did fairly well, so you know, we had like maybe a few patients who initially they weren’t still quite sure of how to approach the phones and so forth. I would say overall maybe 80 percent of the time the patients did excellent. But those who did not do excellent, they improved over time.

Research assistant

As we have noted, EMOCHA intended the phones for intervention use only, and the company put a lock on the devices to prevent personal use. In practice, however, these “locks” were sometimes ineffective, and participants were able to use the phones for personal calls and internet searches. This technical problem—ineffective phone locks—also had unexpected benefits in some cases. Research staff noted that many participants enjoyed having a phone.

There were patients who didn’t have a phone who were really psyched to get a phone. Thought wow, this is like a holiday, oh my gosh. This is amazing. They were excited, they thought it was really decadent and expensive and they felt, wow, I get this phone?

Project director

Among the patient participants interviewed for this study, most reactions were positive. Several noted the superior convenience of the method:

I mean it worked really well for me ‘cause I could just sit at home and take a pill, didn’t have to worry about going in. . . I could just continue with my regular routine and I videotaped it and sent it to [research assistant] and I think I missed one day out of the whole treatment.

Participant 3

I lucked out because I didn’t have to come back every day. . . I got into the video group and that made my life a lot easier, ‘because I wouldn’t have liked going every day to have them watch you take a pill.

Participant 1

The phones also helped patients connect both to study staff as well as to important others in their lives.

The mDOT arm, the reason why I think it [will] be more successful, is because …for participants who don’t have phones or don’t have access to phones, [it’s] a way of communicating not just with research staff but also with the clinic staff and also with family members, friends, because they would use that phone to keep in contact with different people in their circle as well. So, I think that was helpful because it just offered a sort of sense of stability.

Research assistant

And we did have one participant actually who when it was time for his treatment completion, he just said he was very upset because he wouldn’t be coming in once a week and that was the most stable thing that he had in his life … coming in once a week to do weekly pickups with research staff, submit those videos every morning, and that kind of thing, and he was dealing with a lot of instability at that time.

Project director

Another unanticipated benefit of VOT was that it improved the staff’s ability to monitor patients. Because of the daily communications from participants, staff could act quickly in cases of treatment interruption.

And then also with the VOT arm we would check the video and then we could conduct outreach, and for us time does really matter because it depends if they’re in the hospital or if they’re incarcerated. And then sometimes it felt like it was a ticking clock because they need to take their medication, the hepatitis C medication, at a specific time. And so, if they were incarcerated or hospitalized, we needed to get their medication to them as quickly as possible. So, time was of essence.

Project director

However, several participants in our small sample did not like using the phone app. Some mistakenly assumed that the videos were intended to prevent patients from stealing or selling valuable medication.

It was like a trust factor. I don’t think you need to do all that and videotape it. . . I mean, ‘because it is expensive prescription, but it’s not like a mind-altering drug where you can sell it on the street. . . Maybe they did it because it was so expensive. I don’t know the reason behind that.

Participant 4

Other participants felt embarrassed to video what they felt was a private, intimate act.

Taking the video with the app on my phone. I did it, but it was kind of like a little bit, I don’t know, like I did show me swallowing a pill, and I’m really self-conscious… there’s a lot of video that exists of me being goofy and all kinds of other stuff… so I just added it to the whole list of, you know, otherwise embarrassing or otherwise not my best (light) photos . . .

Participant 5

Yeah, having to record myself taking the medication every evening, I just, you know, I don’t know, I didn’t like doing it. ... I didn’t feel like putting myself on camera and sitting and doing the same thing every evening, you know. It was taking pills, you know, just taking pills.

Participant 3

Participants who lacked stable housing did not have privacy in which to record videos:

I have had a couple of patients choose not to sign up because they were worried about the privacy, doing it in a group home setting and perhaps being seen by other clients doing this. . . I had a couple really good candidates not follow through because of that.

Principal investigator

Staff respondents reported that a few of the participants discontinued treatment because they did not want to use the phone for VOT:

We even had one person who felt so strongly about it but who also wouldn’t commit to coming into the clinic that he took three days of pills, was (totally frustrated), turned in his phone and his medication and withdrew from the study….

Project director

### Patient-Level Barriers to Implementation

A major theme among study staff was that the success of the VOT intervention depended on patient characteristics. Several project staff observed different rates of VOT uptake and adherence between patients from different recruitment sites. For example, according to some of our research staff participants, patients recruited from inpatient units or syringe service program sites were more likely to lack access to care and secure housing. Among participants recruited at these sites, overall engagement in the project was more difficult than among participants recruited from other sites.

So we met several of these patients [in the hospital and] signed them directly up. . . But then they often did not follow through or had such severe situations that a trial didn’t really work out for them. And so, for instance, one patient just lost two phones, I mean, and it was just very difficult to picture him making it through. But we tried our best and we got him actually a couple of doses, but he rapidly just you know a day or two goes by he loses a phone, we replace it, he loses another one. . . And those are the types of stories from the inpatient side.

Principal investigator

At another site, the project director noted that she had experienced considerable difficulties at the beginning of the project. At that time, she and her team were trying to recruit HERO participants from a drop-in clinic. Patients at this site had many problems with their phones. When the team stopped recruiting from this site and put their energies into recruiting from a specialty clinic, they found that the patient population was different.

These patients at the specialty clinic] are still actively injecting, but they tend to be not struggling so much. They are more or less stable if they make it all the way to the specialty clinic. . . and they had their own smart phones and that kind of eased things up a little bit.

Project director

Thus, our data suggest that differences in patient populations impacted VOT fidelity. The challenge was not drug use per se. Rather, both structural challenges in patients’ lives—particularly homelessness and other sequelae of poverty, as well as a lack of technological facility—reduced adherence to the phone intervention. Sites that changed their recruitment sites in order to recruit younger, less poor, less impaired patients reported that they saw improved implementation of VOT.

## Discussion

### Summary

Perceptions of the VOT treatment delivery intervention a diverse stakeholder sample that included both study staff and study participants from 8 different sites around the country. Overall, the results suggest a mixed picture for the VOT intervention with respect to implementation, fidelity, and stakeholder experiences. Both staff and patient participants reported positive experiences with VOT. However, the number of lost, stolen, and broken phones was a notable barrier. Patient-level barriers included a lack of technical skills and severe structural constraints such as homelessness. Concerns over surveillance and a lack of privacy were another potential barrier.

### Comparisons to Prior Work

VOT has been used, to date, mainly in tuberculosis treatment. Consequently, most research to date has examined VOT in the context of patients with tuberculosis [19-23]. A recent review found high rates of patient adherence and acceptability with VOT, along with lower costs [24].

However, many studies describe important barriers to implementation, including intensive staff engagement [26], poor technical skills, and most importantly, low phone ownership [27,28]. This report describes the first qualitative study to evaluate VOT implementation for HCV treatment among persons who inject drugs.

### Principal Findings

Our findings reflect some of the themes identified in the prior literature focused on VOT among persons with tuberculosis. A major difference in this study, however, was that we circumvented the phone ownership barrier by providing phones to participants who needed them. This innovation had several consequences. One consequence was prevalent misuse of phones. Although the company had placed a lock on the phones to prevent nonstudy use, some patients were able to circumvent the locks. We also heard many reports of phone breakages and missing phones. Many of the latter were losses, and others may have been stolen. An unexpected consequence of this phenomenon was that some patients became upset when they were unable to prevent the loss of their phones. One participant in our sample reported dropping out because of the shame over losing the phone.

On the positive side, some staff reported that when patients who had been given phones were easier to contact and better able to maintain their own social networks and sources of support, compared to patients without phones. This finding raises the hypothesis that providing phones may constitute an intervention in itself.

Despite this possible advantage, most staff reported that it was much easier to implement the intervention with patients who had their own phones—in part because such patients were more tech savvy and less burdened by economic and housing problems. Across the board, staff reported that the intervention was difficult to implement among older participants, those with a lack of technical skills and participants facing problems such as homelessness.

To make the VOT intervention work, research staff took on the role of reviewing videos. A benefit of this intervention was that staff were able to deepen their connection with patients and deliver useful services. While this was a positive development, it also raises concerns about external validity—a particular concern in a pragmatic trial, which was designed to mimic real-life conditions. Whether such an intervention would work in a setting where the responsibility of checking videos and monitoring adherence was rested with busy clinical staff is unclear.

An interesting finding that emerged in our study was the difference between patients’ original preferences and their actual experiences with the phone. The vast majority of participants offered a choice of VOT or in-person DOT chose the former, due to its superior convenience. This reflects findings in the literature on VOT for tuberculosis, suggesting that many patients show initial enthusiasm for the method. Yet, initial enthusiasm does not mean that patients will be able to use the approach successfully. Although the patient participants in our study chose VOT over DOT, several reported difficult experiences.

### Implications for Users

Our results may provide helpful information for those considering the use of DOT modalities. Treatment may be best provided via multidisciplinary teams that include social workers and other community and government service providers. Patients impacted by social and structural determinants of health may be at risk of failure without such support.

Beyond this obvious point, an important consideration in deciding whether to implement VOT is the question of whether patients experiencing the impacts of SDH will be able to benefit. In a recent evaluation of VOT for tuberculosis patients in New York, Bommakanti et al [32] note that older participants in their study, those with lower education, and those with very low incomes were least likely to own a phone. These authors discuss the implications of requiring smartphones for health interventions, asking the question “who do we leave out?” when imposing such requirements [32]. The results of our study, similarly, suggest that the app may be unsuited to older, less tech-savvy participants—patients who, ironically, may be particularly in need of adherence support. Our findings suggest that VOT should be offered to patients as a choice among several treatment options, and clinicians should be ready to offer new options if patients are not succeeding with VOT.

### Strengths and Limitations

Strengths of our study included (1) a diverse sample, which provided insights from both patients and research staff—2 groups of stakeholders whose experiences were quite distinct—and (2) qualitative methods, which allowed for a deeper exploration of complex experiences.

There were several limitations to the study. First and foremost was the use of a nonrepresentative sample. Exploratory qualitative studies generally do not aim for representative samples. However, for reasons previously explained, the participants interviewed in the study may have been more engaged with treatment, had stronger relationships with project staff, and were easier to contact than typical HERO participants.

### Future Research

Our study findings suggest both the promise and potential problems with VOT for persons who inject drugs treated for HCV. The diverse experiences of staff and patients reported in this qualitative study suggest hypotheses for future research, particularly regarding the role of housing, poverty, low technical literacy, and social support in adherence. Findings also suggest the importance of a formal test of VOT for persons who inject drugs being treated for HCV in a randomized controlled trial. Whether technical skills should be a criterion for offering VOT might be a consideration in such a trial. This future study should provide important information regarding the relative benefits of VOT compared to conventional DOT and other adherence interventions. The contactless method of VOT may be particularly relevant in a post pandemic context. Assessing the benefits of adherence support interventions will be important for improving adherence to highly effective HCV treatments among persons who inject drugs.

## Appendix

Multimedia Appendix 1 Patient interview guide.

Multimedia Appendix 2 Research staff interview guide.

Multimedia Appendix 3 Video-observed therapy study codebook.

## Abbreviations

DOT: directly observed treatment
HCV: hepatitis C virus
HERO: Hepatitis C Real Outcomes
mHealth: mobile health
OTP: opioid treatment program
VOT: video-observed therapy

## Data Availability

Due to the small size of the research staff sample, making our data available to other researchers might entail an unacceptable risk to the confidentiality of participants. Therefore, data from this study will not be made available.
