# Supplementary material for: A Video-Observed Treatment Strategy to Improve Adherence to Treatment Among Persons Who Inject Drugs Infected With Hepatitis C Virus: Qualitative Study of Stakeholder Perceptions and Experiences
Source: J Med Internet Res. 2023 Jun 2;25:e38176. doi: 10.2196/38176 (PMC10276322; doi:10.2196/38176)
Supplement: Multimedia Appendix 1 [file jmir_v25i1e38176_app1.docx]

T2—12 Weeks Post Baseline

Brief Treatment Experiences Interview

*Thank you for answering all these questions. Now I’d like to speak with you about your experiences with the HERO program. Your experiences and feedback as a patient and participant are extremely valuable. This part of the interview will take about 30 minutes. I will be taping your answers on this interview form. Please bear with me if I have to stop you occasionally to get down what you say. The goal of this interview is to understand your perceptions of Project HERO. Ready*?

1. Tell me about your overall experiences with the HERO HepC treatment project.
   1. How did you hear about HERO?
   2. What made you decide to participate in HERO? *(Presumably the patient will say something about needing treatment for HepC)*
      1. Why did you decide to seek treatment for HepC at this time?
      2. Had you thought about getting treatment for HepC before? If yes, what held you back? What barriers did you encounter?
      3. What did you hear about the program that made you want to participate?
   3. Was the program what you expected? Why or why not?
   4. What did you like best about HERO? Why?
   5. What was your least favorite part of the program? Why?
   6. Tell me about your relationship with your patient navigator (if relevant).
   7. Tell me about your experiences with DOT/VOT (if relevant).
      1. Pick up schedule—was it convenient?
2. How well did the HERO program work for you?
   1. Were you able to complete the treatment?
   2. What challenges did you face in participating in the program?
      1. Taking all the meds? Tell me about that.
      2. Getting to the appointments/meeting the navigator? Tell me about that
      3. Participating in research interviews like this one? Tell me about that.
      4. Other…
3. Thank you. Now...in this part of the interview, I want to ask you a little bit about yourself. As you think back on the past year or two, what do you think have been the biggest challenges or hurdles facing you in your life? *Prompt with the following : “please tell me about”:*
   1. Family issues
   2. Health issues
   3. Addiction
   4. Money problems
   5. Housing problems
   6. Other…
4. Thanks for telling me about that. (One by one, work your way through the problem that the person described above):
   1. Now I’m going to ask you about each of these issues. Do you feel that . . . had an impact on your ability to fully participate in HERO? Tell me about that.
5. OK. Now I’d like to ask you about strengths and resources you have in your life. What helps you get through?
   1. Family—tell me about that
   2. Friends—
   3. Work
   4. Spiritual
   5. Personal strengths
   6. Other
6. Thanks. Did any of these supports/resources help you get through the HERO program and treat your HepC successfully?
7. In addition to treating HepC, did the program help you in other ways? How?
   1. Substance abuse treatment, medical treatment for other conditions, help with depression, social services, other
8. Thank you so much. Now, I’d like to ask you how we could improve our program for other participants like yourself. How could we make the program better?
   1. Was there support or help that you would have liked but did not get?
   2. Was it hard to get to appointments or complete the treatment?
   3. Any other challenges that we could have helped with?
9. Is there any other feedback you could give me that might help us improve the project now or in the future?
