# Supplementary material for: A Video-Observed Treatment Strategy to Improve Adherence to Treatment Among Persons Who Inject Drugs Infected With Hepatitis C Virus: Qualitative Study of Stakeholder Perceptions and Experiences
Source: J Med Internet Res. 2023 Jun 2;25:e38176. doi: 10.2196/38176 (PMC10276322; doi:10.2196/38176)
Supplement: Multimedia Appendix 2 [file jmir_v25i1e38176_app2.docx]

PROJECT Staff INTERVIEW GUIDE

Questions for the EMOCHA Analysis

*Thank you for doing this interview with me. It will take about 15- 30 minutes. I would like to tape the interview with your permission.*

Basically, I’d like to find out how things are going!

How are things at your CHC/Community site(s)?

- - What’s going well?
  - What challenges are you facing?
  - What are you doing to cope with those challenges?

How are things at your OTP site(s)?

- - What’s going well?
  - What challenges are you facing?
  - What are you doing to cope with those challenges?

Great. Now I’d like to run over a few of the issues that have come up in other interviews/meetings:

- What patient issues are challenges at your site?
- How are the EMOCHA phones working at your site?

How are you coping with these challenges? It would be really helpful to understand the specific strategies you are using to address problems that have come up.

Given your overall experiences with the project so far, what do you think are the implications for sustainability/replication of the study interventions?

Keeping in mind both what is going well, as well as the challenges you are facing, what would you do differently next time?

Anything else you can tell me? Anticipate any new issues coming up down the line?

THANKS!!!
