# Supplementary material for: A Video-Observed Treatment Strategy to Improve Adherence to Treatment Among Persons Who Inject Drugs Infected With Hepatitis C Virus: Qualitative Study of Stakeholder Perceptions and Experiences
Source: J Med Internet Res. 2023 Jun 2;25:e38176. doi: 10.2196/38176 (PMC10276322; doi:10.2196/38176)
Supplement: Multimedia Appendix 3 [file jmir_v25i1e38176_app3.docx]

| **STAFF STUDY CODES** |
| --- |
| Barriers to Implementation |
| VOT challenges |
| Loss & breakage |
| Technical issues |
| Misuse |
| Patient difficulties |
| Patient Barriers |
| adherence |
| Differences in HepC Infected Groups |
| disruptive behaviors |
| Eligibility Issues |
| Health Issues |
| Loss to follow up |
| Shame |
| Other patient level barriers |
| Facilitators of Implementation |
| VOT Benefits |
| Improved monitoring |
| Having a phone |
| Improved relationship |
| Connection/network |
| Adherence |
| Patient Motivation |
| Providing support/services |
| Suggestions for improvement |
| Surprises |
| **Patient Study Codes** |
| Enrolling in HERO |
| Barriers |
| Facilitators |
| Reasons for Enrolling in HERO |
| Experiences with the program |
| Benefits of Program |
| Burden or HERO requirements |
| adherence |
| Attending appointments |
| Data Collection |
| Interactions with Clinical Team |
| Interactions with the Research Team |
| Patient Assessment of HERO |
| External Challenges |
| External Facilitators |
| Internal Strengths |
| Internal Weakness |
| My Personal barriers |
| Attitude |
| Drug Use |
| Shame |
| Medical and Health |
| Motivation |
| Personal Strengths |
| Spirituality |
| Support system |
| External (Structural) Barriers |
| Economic |
| Housing |
| Money Issues |
| Prison |
| Rehab |
| Transportation and Distance |
| Work schedule |
| Suggestions for improvement |
| VOT Patient Codes |
| Video experiences |
| Interactions with staff |
| Picking up meds |
| Having a phone |
| Technical problems |
| Loss of phone |
